# A mixed methods approach to developing and evaluating oncology trainee education around minimization of adverse events and improved patient quality and safety

**DOI:** 10.1186/s12909-016-0609-1

**Published:** 2016-03-12

**Authors:** Anna Janssen, Tim Shaw, Lauren Bradbury, Tania Moujaber, Anne Mette Nørrelykke, Jessica A. Zerillo, Ann LaCasce, John Patrick T. Co, Tracy Robinson, Alison Starr, Paul Harnett

**Affiliations:** Research in Implementation Science and e-Health Group (RISe), Faculty of Health Sciences, Sydney West Translational Cancer Research Centre, The University of Sydney, Sydney, NSW Australia; Research in Implementation Science and e-Health Group (RISe), Faculty of Health Sciences, The University of Sydney, Sydney, NSW Australia; Crown Princess Mary Cancer Care Centre, Westmead Hospital, Westmead, NSW Australia; Dana-Farber Cancer Institute, Boston, MA USA; Partners HealthCare, Boston, MA USA; Faculty of Health, Disciplines of Nursing and Midwifery, University of Canberra, Canberra, ACT Australia; Sydney West Translational Cancer Research Centre, WestmeadHospital, Sydney, NSW Australia; Western Sydney Local Health District, Westmead, NSW Australia

## Abstract

**Background:**

Adverse events are a significant quality and safety issue in the hospital setting due to their direct impact on patients. Additionally, such events are often handled by junior doctors due to their direct involvement with patients. As such, it is important for health care organizations to prioritize education and training for junior doctors on identifying adverse events and handling them when they occur. The Cancer Cup Challenge is an educational program focuses on quality improvement and adverse event awareness targeting for junior oncology doctors across three international sites.

**Methods:**

A mixed methodology was used to develop and evaluate the program. The Qstream spaced learning platform was used to disseminate information to participants, as it has been demonstrated to impact on both knowledge and behavior. Eight short case based scenarios with expert feedback were developed by a multidisciplinary advisory committee containing representatives from the international sites. At the conclusion of the course impact on participant knowledge was evaluated using analysis of the metrics collected by the Qstream platform. Additionally, an online survey and semi-structured interviews were used to evaluate engagement and perceived value by participants.

**Results:**

A total of 35 junior doctors registered to undertake the Qstream program, with 31 (88.57 %) successfully completing it. Analysis of the Qstream metrics revealed 76.57 % of cases were answered correctly on first attempt. The post-program survey received 17 responses, with 76.47 % indicating cases for the course were interesting and 82.35 % feeling cases were relevant. Finally, 14 participants consented to participate in semi-structured interviews about the program, with feedback towards the course being generally very positive.

**Conclusions:**

Our study demonstrates that an online game is well accepted by junior doctors as a method to increase their quality improvement awareness. Developing effective and sustainable training for doctors is important to ensure positive patient outcomes are maintained in the hospital setting. This is particularly important for junior doctors as they are working closely with patients and learning skills and behaviors, which will influence their practice throughout their careers.

## Background

The rate of adverse event reports in hospitals globally has remained consistent over the last 20 years [1, 2], which is particularly disappointing given their direct impact on patient quality care. Adverse events are usually defined as incidents that result in harm to a patient–for example, either a short-term or permanent disability or in extreme cases patient death. Not only do adverse events have a severe impact on the patient, but the literature suggests that as many as one third of these events are preventable [3]. Hospital-based adverse events cover a wide range or of incidents, many of which can be classified as due to either systemic or human factors [4, 5], and negatively impact on patient quality of care.

Doctors in training represent a group of health professionals that maybe particularly vulnerable to involvement in adverse events due to their high patient contact, workload and relative inexperience [6]. This is evident in a number of studies that have reported on a phenomena referred to as the ‘July Effect’ where there have been reported increases in adverse events early in the academic training year [7].

Effective education and rigorous training has been recognized as an important component of developing safe and efficient health professionals. This is reflected by organizations such as the Accreditation Council for Graduate Medical Education (ACGME) mandating that residency programs provide education for residents and fellows in safety and quality [8]. Quality improvement has emerged as an increasingly important focus of activity in health services and it is essential that clinicians understand the effectiveness of such interventions [9]. In addition, current international training curricula include key competencies in safety and quality [10]. This highlights the need for guidance on how to design and research quality improvement and safety programs. In spite of this focus, the delivery of effective education to health professionals in training remains a key challenge and under-researched area.

Physicians in training are required to demonstrate skills and knowledge across an increasingly large number of competencies while developing skills in their chosen discipline [11, 12]. Even though adult learning principles have long identified that education and training must be contextually relevant and directly linked to practice [13], many safety and quality programs in hospitals are delivered via didactic lectures or workshops that are not specific to the training environment or practice [14]. While online learning is increasingly employed for the scalable delivery of education to doctors in training, there is little evidence in the literature regarding whether computer-based online educational methodologies are effective in actually changing behavior. Furthermore, if online programs are to include education and training in safety and quality to meet mandatory training requirements then it is essential that researchers can demonstrate evidence of their impact.

Qstream is a novel, evidence-based form of online education that has been demonstrated to improve knowledge acquisition and recall [15]. It has also been repeatedly demonstrated to change participant behavior and maintain that change over time [16, 17]. Participants in Qstream courses receive repeating, short, case-based multiple choice questions with feedback via email in a reinforcing pattern over a number of weeks. The methodology is based on two core psychological research findings: the spacing and testing effects. The *spacing effect* refers to the finding that educational encounters that are repeated over time increase the acquisition and retention of knowledge. The *testing effect* refers to the finding that the process of testing does not only measure knowledge, but also improves retention [18, 19]. It has further been demonstrated that participants find repetition of cases via email effective for completing the course due to the ease of completing cases in existing schedules [16].

In a randomized trial this Qstream platform was found to increase learning efficiency by over 35 % over a non Qstream program with identical content. In addition, Qstream includes a gamification element whereby participants can compete as both individuals and teams and review their progress against de-identified leader boards [20]. Gamification refers to the use of individual game mechanics such as leader boards in order to enhance another online tool such as an online course. The use of gamification can be beneficial in online education as it has the ability to both engage and motivate learners, strengthen social ties and preserve the original focus of the learning activity [21]. The use of gamification elements in e-learning has also been demonstrated to result in more accurate results on quizzes due to the use of competition, which stimulates learners to interact more with their peers and the content of the course [22].

This study evaluated the feasibility and acceptability of using Qstream to deliver safety and quality education in an oncology-specific context to doctors in training in the United States, Australia and Denmark. In particular, the program focused on frequently encountered urgent clinical scenarios, adverse events from cancer treatments, and event reporting of errors and near misses to demonstrate key learning points. The study also evaluated the impact of competition in motivating participation through promoting the program as a friendly competition between the countries titled the ‘Cancer Cup Challenge’.

## Methods

### Setting

The aim of the Cancer Cup Challenge was to develop a program targeting key safety and quality issues that are encountered by physicians training in medical oncology. An expert Advisory Committee was convened in March 2014 to oversee the development of the program. This Committee consisted of senior medical oncologists, quality improvement staff, educational designers, senior oncology registrars and research fellows across participating sites.

The program was developed by representatives from international sites in Australia, Denmark and The United States of America (USA). This included individuals from the Sydney West Translational Cancer Research Centre in Sydney, Australia; Aarhus University Hospital in Denmark; and the Partners Program in Boston, the United States of America.

### Curriculum development

**Fig. 1:**
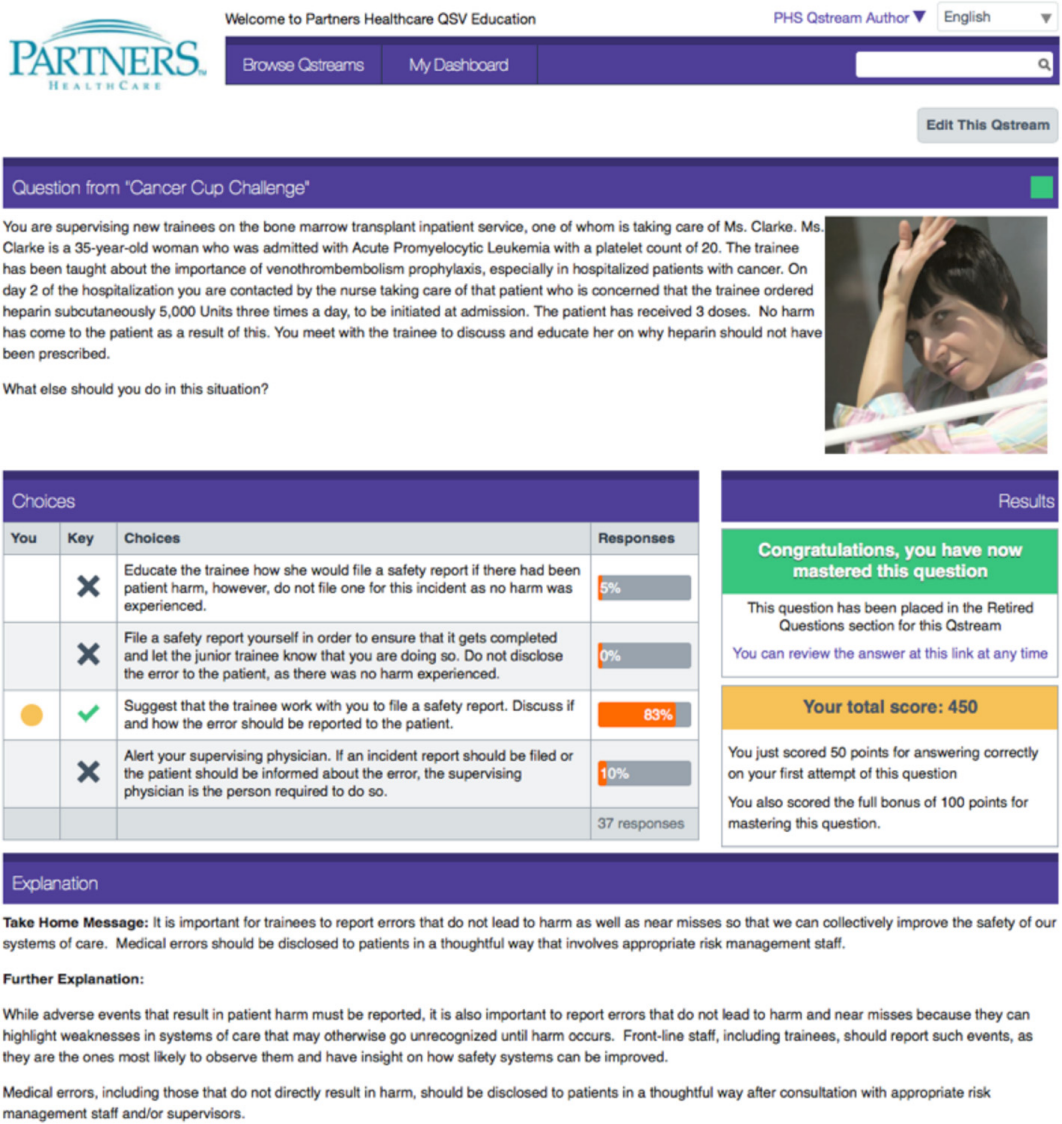
An example Qstream case from the Qstream: Cancer Cup Challenge

The Advisory Committee oversaw the development of a set of key learning objectives which were turned into one sentence take-home messages to be used for the Qstream cases. These key take-home messages related to specific practice points on safety and quality for oncology patients. They included items such as fertility preservation and drug interactions, particularly in regard to oral chemotherapy. The take home messages were derived from a number of sources including: a review of the literature; a review of local adverse events or near-miss reports at each site and 2 consensus building workshops attended by senior oncologists and senior oncology trainees in Australia and the United States. An initial set of 34 key take home messages was developed that was ultimately refined to eight through an interactive review process by the Advisory Committee. *Refer to* Fig. 1*for an example Qstream case from the Cancer Cup Challenge*.

Once the take home messages had been identified, the Advisory Committee worked collaboratively to write short clinical case scenarios that reinforced the specific messages. The correct answer was identified and input as a multiple choice response. This was augmented by three distractor options which could be equally plausible to a participant. The final stage of building a case was developing the expert feedback. This expanded on the take home message in more detail and provided additional resources for further reading.

### Participant recruitment

Junior oncology trainees were recruited to participate in the study from the Basic Science in Oncology Course (BSOC) in Australia, Aarhus University Hospital in Denmark and the Partners Program in the United States of America in October 2014. The research team was provided with contact details for a recruitment coordinator at each site. This coordinator was provided with generic text to create an expression of interest email for participants at their site. This was emailed to all medical oncology trainees at the site. Once recruitment had been completed participants were allocated to teams based on their country of origin and then were emailed a link to access the Qstream spaced learning system on which the Qstream: Cancer Cup Challenge was running by a member of the research team. The research team did not give participants access to any patient safety or dverse events training prior to undertaking the Qstream: Cancer Cup Challenge.

### Course administration

Once participants enrolled in the course cases were emailed to them and could be completed on a personal computer, tablet or smart phone. The cases were sent to participants in the following spaced manner:

- Each participant received an email every 2 days containing at least 2 cases
- If they answered a case incorrectly, it was re-sent 5 days later
- Once a case was answered correctly it was retired
- The course was completed once all questions had been retired.

Each Qstream case consists of an evaluative component (a clinically-relevant multiple-choice question) and an educational component (the correct answer and a detailed explanation of the answer). Participants submit an answer, receive immediate feedback, and compare their performance with peers. To harness the educational benefits of the spacing effect, the case is repeated until the participant answers the case correctly. The decision to repeat only incorrect cases was made based on data from previous Qstream programs at the clinical sites. This data indicated that repeating correct cases increased the likelihood participants would not complete the program.

During the Cancer Cup Challenge participants were allocated to teams to evaluate the impact of the gamification element. Upon answering a question, participants were presented with a de-identified league table that indicated how they were performing compared to their peers. Participants were sent periodic emails containing team scores.

### Analysis

Mixed methods were used to evaluate the impact of the Cancer Cup Challenge on participant knowledge, skills and team engagement. Quantitative data collected by the Qstream system was analyzed for participant response accuracy. Qualitative data was collected through semi-structured interview and online survey to assess participant experience of the program regarding content and format. Qualitative data from interviews was transcribed and thematically analyzed.

Upon completion of the Cancer Cup Challenge participants were emailed a link to the online survey. This survey asked them to rank aspects of the course format and course content using a Likert ranking scale. They were also given the opportunity to leave free-text comments on their views of the course. A reminder to complete the survey was emailed out 1 week after the initial survey link was disseminated.

Participants were also invited to participate in a brief interview within 4 weeks of completing the course to explore the perceived impact of the Qstream education program on their knowledge and behavior in relation to adverse events in cancer care. Interviews were conducted over the phone or in person and took between 10 and 20 min to complete. The interviews were semi-structured without set questions. However, an interview guide was developed to maintain a consistent structure for all the interviews. Interviews first explored registrar and fellow needs in regards to adverse events training and how the Qstream program engaged participants and impacted on their educational experience.

Permission to conduct this study has been received from the Western Sydney Local Health District Human Research and Ethics Committee.

## Results

### Participation and case performance

Of 50 medical oncology trainees invited to participate across the three sites, 35 registered for the course. Thirty one (88.57 %) of these participants went on to successfully complete each case presented via the Qstream platform. Overall, 35 participants answered at least one question (70 % of those invited and 100 % of those who enrolled), and 31 participants completed the program (62 % of those invited and 89 % of those who enrolled). The demographic breakdown for the 35 participants was:16 male (46 %), 15 female (43 %) and four (11 %) did not indicate a gender. *Refer to* Table 1*for a comparison of participation based on country. Refer to* Table 2*for a gender distribution of participants by country*.

**Table 1:** Details of the number of participants who enrolled in the Qstream: Cancer Cup Challenge compared to the number who completed

Country	Number enrolled	Percentage enrolled	Number completed	Percentage completed
Australia	12	75 %	11	92 %
Denmark	11	79 %	11	100 %
The United States of America	12	63 %	10	83 %

**Table 2:** Demographic breakdown of the number of participants enrolled in the Qstream: Cancer Cup Challenge based on nationality

Country	Male participants	Female participants	Unspecified gender
Australia	6	4	2
Denmark	4	6	1
The United States of America	6	5	10

**Table 3:** Thematic overview of cases compared to percentage of participants who answered correctly on first attempt

Case take home message	Total % correct responses on first attempt combined	Average number of attempts required to retire case	Minimum number of attempts to retire case	Maximum number of attempts to retire case
For patients at home, toxicities are serious and must be monitored and managed as closely as any reactions observed in the clinic or hospital. Ipilimumab can result in an autoimmune phenomenon that requires treatment with steroids.	64.29 %	1	1	4
Fertility preservation should be discussed with all patients of child bearing age, including sperm banking which should be offered whenever possible before a young man begins chemotherapy.	67.44 %	1	1	2
Spinal cord compression is an emergency and requires neurosurgical or radiation intervention.	71.05 %	1	1	2
Patients may not volunteer that they are not taking their medications as prescribed. Physicians should work with ancillary and nursing services to address these issues as low adherence can cause poorer clinical outcomes.	71.79 %	1	1	2
It is important for trainees to report errors that do not lead to harm as well as near misses so that we can collectively improve the safety of our systems of care. Medical errors should be disclosed to patients in a thoughtful way that involves appropriate risk management staff.	78.38 %	1	1	3
Drug/drug interactions are vital to review when prescribing oral chemotherapy. Capecitabine can potentiate warfarin levels and result in life-threatening bleeding.	78.95 %	1	1	2
Recognize the inappropriate, appropriate and required indications for use of growth factor support.	87.88 %	1	1	1
Due to the risk of life-threatening infection, it is vital to immediately treat neutropenic fever. Steroids can mask a fever so clinical judgment must be used to watch for signs/symptoms.	100 %	1	1	5

76.57 % of cases were answered correctly on the first attempt. However, only one case was answered correctly by all participants on the initial try. All the others had at least two participants answer incorrectly on the first attempt. One case had significantly more incorrect answers than all others with seven participants requiring two or more attempts to retire it. The case explored the challenges of monitoring toxicities that cancer patients experience at home.

Case performance was also reviewed across the three international teams. All teams had three cases which all participants answered correctly on the first attempt. However, only one case was answered correctly by all teams on the first attempt, the case dealt with treating neutropenic fever. In Addition, the Australian team scored 100 % on the first attempt for two cases, one on spinal cord compression, and one on oral chemotherapy drug interactions. The Danish team scored 100 % on the first attempt for a case on error reporting for trainees, and one on appropriate use of growth factor support. The US team scored 100 % of on the first attempt for a case on poor patient medication compliance, as well as a case on managing toxicities in patients as closely as would be done if observed in the hospital setting. *Refer to* Table 3*for an overview of the percentage of participants who answered each case correctly on first attempt compared to case take home message*.

Four participants who begun undertaking the Qstream course, but did not complete it. Of this group one participant answered three cases before discontinuing, two participants completed four cases before discontinuing, and one participant answered seven cases before discontinuing. One participant did not answer a single case incorrectly before discontinuing the course, with the other three only answering one case incorrectly. Only one participant discontinued the course directly after answering a case incorrectly, the remaining three discontinued after answering a case correctly. Two participants that discontinued the course answered the same case incorrectly, a case which related to oral chemotherapy interactions.

A representative of the research team contacted each of the four participants who did not complete the course to find out why. Unfortunately, none of the non-completers responded to this contact.

### Participant experience

**Fig. 2:**
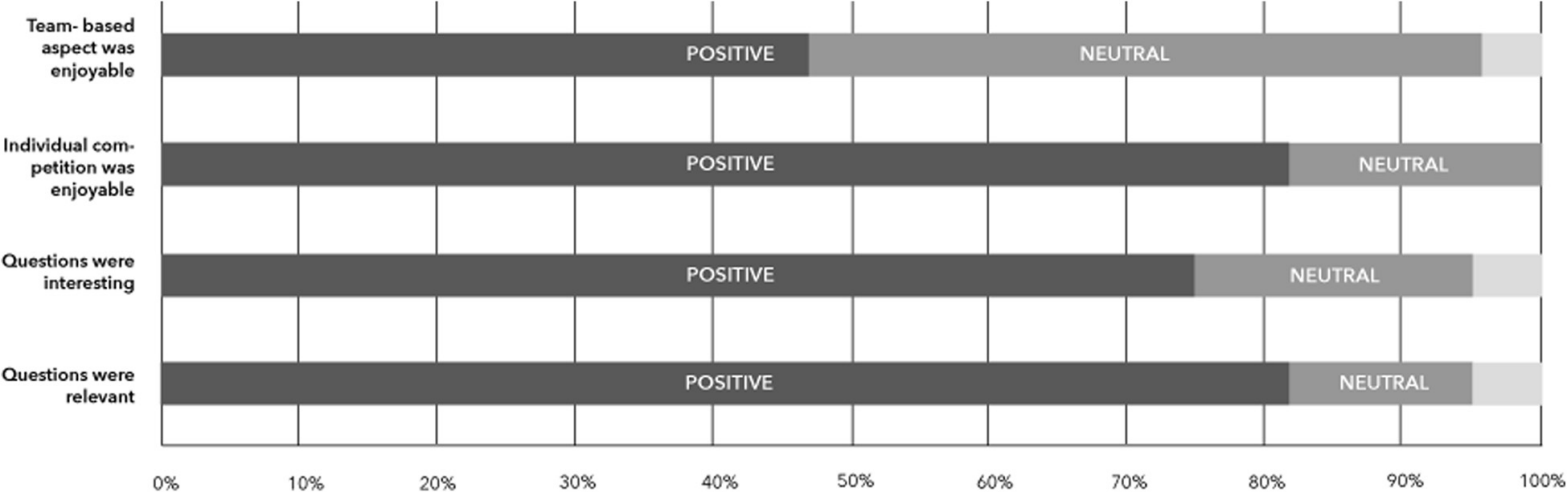
A visualization of Likert responses showing participant engagement in the Qstream: Cancer Cup Challenge

At the conclusion of the Cancer Cup Challenge all participants were asked to complete a brief online survey. Seventeen participants responded to this request and completed the online survey. Participants were asked to rate a range of questions on the content and structure of the course using a 1–5 scale, where 1 was lowest agreement and 5 represented highest agreement. A majority of respondents, 76.47 %, agreed that the cases for the course were interesting. Additionally, 82.35 % of respondents felt the cases were relevant to them. In regard to the format of the course, 47.05 % of respondents indicated that they enjoyed the team-based aspect of the course. In contrast 82.36 % of respondents indicated they enjoyed the individual competition. *A visualization of these Likert responses is provided in* Fig. 2.

Participants were also given the opportunity to leave free-text comments regarding strengths and weakness, along with any general comments about the Cancer Cup Challenge. There were 32 free-text comments about the Cancer Cup Challenge, with some respondents leaving more than one comment. Of those, 21 comments indicated the course was a generally positive experience. Reasons respondents enjoyed the course included the expert feedback on cases, the competitive aspect of the course, and the flexibility and manageability of the course format. One respondent indicated they appreciated that the cases covered scenarios that were less commonly considered issues in education and training.

Of the participants who did not find the course beneficial, the most commonly cited reason was that the cases were too easy. This was closely followed by respondents stating they would prefer receiving more cases.

### Interview thematic review

A total of 14 participants volunteered to participate in semi-structured interviews about the Cancer Cup Challenge. Of the 14 interviewees a total of five were from Australia, two were from Denmark and 12 were from the United States of America. Once interviews had been conducted they were transcribed, de-identified and then content reviewed to identify key themes. A total of five broad themes emerged from the analysis: Impact of course on knowledge and confidence, enjoyment and engagement, motivation for course completion, online learning/Qstream format, and course content in general.

Of the 14 participants interviewed nine specifically commented on the level of engagement and enjoyment of the course. All of these respondents indicated that they found the course engaging and enjoyable. Three participants noted that this was due to the format itself, the below being representative of these comments:

“*It was engaging. It didn’t take very long to do so it wasn’t a burden.*”

A further two participants made comments stating their engagement and enjoyment of the course was linked to the content. One participant specifically noted that the Cancer Cup Challenge could have been more engaging with revised content.

The topic of motivation to complete the Cancer Cup Challenge was another theme that emerged from the analysis. Interestingly, respondents were quite divided when it came to their motivation to continue completing cases for the course. Five respondents commented that their primary driver was knowledge acquisition, or interest in the topics covered in the course. Other respondents indicated that the leaderboards and individual competition was a motivator to continue, though not necessarily the primary one.

In regard to the gamified aspect of the course and its impact on their motivation, participants held mixed views. Three respondents specifically indicated that they did not feel the team based competition motivated them to complete the course, but they didn’t think it was problematic either. In contrast, another three respondents indicated they specifically liked this aspect of the course, finding competition to be a significant driver as well as the excitement of competing with teams from around the world. The below quote is representative of this:

“I think even though we didn’t know exactly who was in our team, it was still a good motivation to actually provide a little bit of excitement to the thing rather than just turning it into another exercise we had to do. I think that was certainly a good motivation to complete at least the next case. I quite enjoyed that.”

It should also be noted that there was one interesting outlying response made by a participant. The participant noted that they were motivated by a desire to help evaluate an online tool which may be developed for specialist training in future:

“Primarily it was to do you a favour in a way because the med said you wanted to develop some e-learning and I think that’s very important because if you can develop some good e-earning which can be part of our specialist training that would be excellent.”

Participants also had mixed responses in relation to the impact of the Cancer Cup Challenge had on their knowledge and confidence of the learning points. Several participants indicated the course increased their knowledge and confidence, or had a small impact on reinforcing their current knowledge. Only one participant felt that the course had no impact at all. However, a majority of respondents suggested that much of the case content, whilst interesting, was a little too simplistic for their current level of training. Nevertheless, most participants also rated the program as highly useful and relevant:

“It certainly prompted me to think about some of the alternative pathways that I could’ve taken in each situation and why they might be incorrect or less correct and putting my own best answer into context. I found it useful from that end even though most of the questions I felt I knew exactly which answer I wanted to put down.”

Of the respondents who felt the course impacted on their knowledge, the majority suggested this was because of the applicability of the cases to their clinical practice. Several respondents indicated they had dealt with situations during their training that were covered by the cases. One also added that they could easily see themselves encountering the case scenarios in practice.

Finally, two respondents noted that the course was valuable for illustrating similarities and differences in cancer care globally. One participant noted:

“It was helpful to know that there was a shared terminology globally (across institutions). Everyone is dealing with the same problems internationally and people are working in the same framework. It was nice to see this is what everyone, everywhere is dealing with.”

Interview participants frequently commented on the Qstream format, with all but one finding it appealing. Eleven respondents indicated that flexibility and the ability to answer cases at their convenience was an advantage of the platform. Four respondents made particular note of the fact they completed the course on their smart phones in between other activities. Seven participants specifically commented on the user friendly interface.

Additionally, five respondents mentioned the appeal of the way the Qstream platform disseminates cases. Several noted that the way the system prompted participants to complete cases they may have forgotten about was very useful. One respondent commented:

“I thought it was very good that the prompt came through and it would come through again if you didn’t answer the question. That kind of reminded me that the course was there because you can forget these things.”

The final theme that emerged from analysis of the interviews was generalised comments about course content. Seven participants gave responses regarding content that could be included in future courses, three of whom specifically asked for more cases on chemotherapy errors, drug interactions and other issues. In addition, three respondents made general comments about the length of the course, expressing a desire for there to be more cases.

## Discussion

This study demonstrated the feasibility of Qstream as a delivery vehicle for specialty-specific content in the area of safety and quality. The completion rates for the program, which was entirely voluntary, were encouraging and indicate a high level of engagement by participants in each country. This finding aligns with other studies using Qstream in the context of residency training [20]. Once again, the case-based and bite-sized nature of the program was found to be appealing to busy doctors in training. Feedback from trainees indicates that the use of oncology specific scenarios motivated participation. While this finding is perhaps not surprising, it does demonstrate that junior doctors are interested in using non-traditional training delivery methods such as online course games.

The ability of the gamification element of the program to motivate participation supports the use of friendly competition in health professional training programs and is consistent with findings in other contexts [23]. Howerver, further research is needed to explore using this innovative approach in medical education in the clinical setting as there is no other research on the topic. This is a significant gap due to the busy schedules of health professionals and the difficulty of engaging this group in online education. Incorporation of game based approaches can be used to increase learner motivation to complete an online program, particularly if it is asynchronous, as was demonstrated in this study. The use of international competition clearly motivated a number of participants and the use of high-profile organizations in each country likely enhanced this. However, it was interesting to note that the individual competition appeared a more significant motivating factor than the international team-based competition. This indicates the value in establishing competition just at a program level to stimulate participation.

The use of doctors in training to develop the core cases in this program also provides a mechanism to meet training recommendations from organisations such as the ACGME which mandate direct engagement of residents and fellows in educational activities to enhance high quality patient care [8]. Literature shows that residents and junior doctors may perceive events and actions in unique ways due to their frequent interactions with patients. In particular they may be well suited to identifying near misses and other incidents not normally captured in current reporting structures [6]. Case-based programs in safety and quality such as Qstream engage trainees and serve as effective tools to teach critical topics such as patient safety and quality improvement.

Strengths of the study include that over 50 % of participants completed the feedback survey and 14 participants took part in semi-structured interviews allowing for further valuable assessment of the value of the program. Limitations of the study include the lack of available measures to judge impact on practice and relatively low total number of participants in the study. Although the sample size was small comparative to the number of junior doctors in the clinical setting, medical oncology junior doctors represent a sub-set of trainees which is much smaller. In this study all medical oncology junior doctors at the three sites were invited to participate in this study and there was a high participation of oncology trainees at each site. In future studies it may be beneficial to use a pre and post test approach in order to precisely measure knowledge change amongst participants. Additionally, the inclusion of more hospital sites may be a benefit in increasing the number or individuals eligible to participate in the course.

## Conclusion

Qstream represents a tool that can be used by individuals and organizations to engage junior doctors in patient safety and quality improvement training. The ability of Qstream to motivate participation, combined with its previously demonstrated impact on behaviour, has implications for directors of training programs. Our findings also illustrate the benefits of tailoring content to the specialty-specific context of the trainee, as well as introducing a gamification element to enhance participation.

### Ethics approval and consent to participate

Permission to conduct this study has been received from the Western Sydney Local Health District Human Research and Ethics Committee.

### Availability of data and materials

Data will not be shared due to restrictions stipulated by the ethics committee that approved this study.
